# Improvement of drought tolerance by overexpressing *MdATG18a* is mediated by modified antioxidant system and activated autophagy in transgenic apple

**DOI:** 10.1111/pbi.12794

**Published:** 2017-08-22

**Authors:** Xun Sun, Ping Wang, Xin Jia, Liuqing Huo, Runmin Che, Fengwang Ma

**Affiliations:** ^1^ State Key Laboratory of Crop Stress Biology for Arid Areas College of Horticulture Northwest A&F University Yangling Shaanxi China; ^2^ Department of Genetics, Development and Cell Biology Iowa State University Ames IA USA

**Keywords:** autophagy, apple, *MdATG18a*, drought, reactive oxygen species, oxidized protein

## Abstract

Autophagy is a major and conserved pathway for delivering and recycling unwanted proteins or damaged organelles to be degraded in the vacuoles. AuTophaGy‐related (ATG) protein 18a has been established as one of the essential components for autophagy occurrence in *Arabidopsis thaliana*. We previously cloned the *ATG18a* homolog from *Malus domestica* (*MdATG18a*) and monitored its responsiveness to various abiotic stresses at the transcriptional level. However, it is still unclear what its function is under abiotic stress in apple. Here, we found that heterologous expression of *MdATG18a* in tomato plants markedly enhanced their tolerance to drought. Overexpression (OE) of that gene in apple plants improved their drought tolerance as well. Under drought conditions, the photosynthesis rate and antioxidant capacity were significantly elevated in OE lines when compared with the untransformed wild type (WT). Transcript levels of other important apple *ATG* genes were more strongly up‐regulated in transgenic *MdATG18a* OE lines than in the WT. The percentage of insoluble protein in proportion to total protein was lower and less oxidized protein accumulated in the OE lines than in the WT under drought stress. This was probably due to more autophagosomes being formed in the former. These results demonstrate that overexpression of *MdATG18a* in apple plants enhances their tolerance to drought stress, probably because of greater autophagosome production and a higher frequency of autophagy. Those processes help degrade protein aggregation and limit the oxidation damage, thereby suggesting that autophagy plays important roles in the drought response.

## Introduction

Plants are inevitably challenged by various environmental stresses such as drought, salt, intense irradiance and heat. Because climates are warming and water supplies are becoming limited, drought stress is now a global phenomenon that significantly threatens future crop production (Zhao and Running, 2010). Drought conditions generate and accumulate reactive oxygen species (ROS), leading to membrane disruption, enzyme dysfunction and protein oxidation and aggregation (Tsugane *et al*., 1999). In plant cells, ROS can be scavenged through both enzymatic and nonenzymatic pathways (Apel and Hirt, 2004). The enzymatic detoxification system involves crucial enzymes such as superoxide dismutase (SOD), catalase (CAT), peroxidase (POD), ascorbate peroxidase (APX), dehydroascorbate reductase (DHAR), monodehydroascorbate reductase (MDHAR) and glutathione peroxidase (GR). While the nonenzymatic scavengers include efficient antioxidants such as ascorbate and glutathione (Foyer and Noctor, 2009), these ROS‐scavenging systems are crucial to the plant tolerance under stresses.

Autophagy, a conserved cellular process in eukaryotes, plays an important role in recycling nutrients and cytoplasmic components, as well as in conferring tolerance to biotic and abiotic stresses (Han *et al*., 2015; Havé *et al*., 2016; Mizushima and Komatsu, 2011; Ryabovol and Minibayeva, 2016; Wang *et al*., 2016, 2015b; Xiong *et al*., 2005). In the cells of plants that undergo starvation or other stresses, production of double‐membrane structures, the autophagosomes, is induced. Aggregated proteins or damaged organelles in the cytoplasm are engulfed by the autophagosomes and are degraded for recycle when autophagosomes fuse with the vacuole. Plant autophagy can be activated by various abiotic factors, for example, nutrient starvation, oxidative stress, salinity, drought, heat and hypoxia. These stresses enhance the accumulation of ROS and oxidized proteins. Autophagy‐defective mutants are hypersensitive to those stress conditions (Chen *et al*., 2015; Han *et al*., 2011; Liu *et al*., 2009; Xiong *et al*., 2005, 2007b; Zhou *et al*., 2014b). With reduced autophagic degradation rate, *Arabidopsis* RNAi‐*AtATG18a* plants are hypersensitive to hydrogen peroxide (H_2_O_2_) and accumulate more oxidized proteins than WT (Xiong *et al*., 2007b), indicating that autophagy is important in removing oxidized proteins under oxidative stress. Meanwhile, RNAi‐*AtATG18a* seedlings showed more severe growth inhibition than WT upon drought and salt treatment (Liu *et al*., 2009), implying autophagy as crucial participation in stress response. The *Arabidopsis* autophagy‐defective mutants, like *atg5* and *atg7*, are demonstrated to have compromised tolerance to oxidative stress (Zhou *et al*., 2013). Moreover, rice *Osatg10b* mutants are sensitive to methyl viologen (MV) with high levels of oxidized proteins (Shin *et al*., 2009). Recent study in tomato found that overexpression of a heat‐shock transcription factor *A1a* (*HsfA1a*) confers drought tolerance by up‐regulating *ATGs* expression and inducing autophagosomes formation, which is achieved through the direct binding of HsfA1a to the promoters of *ATG10* and *ATG18f*. These also suggest that autophagy functions in promoting plant survival under a water deficit (Wang *et al*., 2015b).

In yeast, the WD40 repeat‐containing protein Atg18 is able to bind phosphatidylinositol 3‐phosphate [PtdIns(3)P] and phosphatidylinositol 3,5‐bisphosphate [PtdIns(3,5)P_2_] (Krick *et al*., 2006). The PtdIns(3)P binding capacity of Atg18 is needed for efficient recruitment of Atg8 and Atg16 during phagophore formation at the phagophore assembly site (PAS) (Nair *et al*., 2010). The presence of Atg18/21 complex blocks access of Atg4 to Atg8‐phosphatidylethanolamine (PE) at PAS, preventing a premature cleavage event. However, following completion of the autophagosome, probably under regulation of Atg1, the Atg18/21 complex will dissociate and allow Atg4 to cleave Atg8 from PE. Therefore, the key aspect of post‐translational regulation of autophagy by Atg4 is closely related with Atg18/21 (Nair *et al*., 2010). Yeast *Atg18* gene is required for starvation‐induced autophagy, as *atg18* mutant is unable to accumulate autophagosomes in response to starvation (Barth *et al*., 2001; Guan *et al*., 2001). *Arabidopsis* has eight *ATG18s* homologs (a‐h), and each member shows a different transcript pattern. Only *AtATG18a* showed a significant increase in transcript level in both sucrose and nitrogen starvation conditions, as well as during artificial senescence and oxidative stress treatment (Xiong *et al*., 2005, 2007a). As stated earlier, RNAi‐*AtATG18a* plants, with defect in autophagosomes formation, are sensitive to oxidative stress, high salt, drought and necrotrophic pathogens (Lai *et al*., 2011; Liu and Bassham, 2012; Liu *et al*., 2009; Xiong *et al*., 2005). On the account of importance of ATG18a in autophagosomes formation and roles in stress response, we carried out our study on autophagy in apple plants. *MdATG18a* is the first autophagy gene cloned from apple (*Malus domestica*), and little is known about the function of autophagy in apple plants towards environmental stresses. We found that *MdATG18a* has conserved WD40 domains and is transcriptionally induced by various abiotic stresses including drought (Wang *et al*., 2014). As one of the most economically important fruits in the world, apple production faces great challenge towards drought stress in Loess Plateau, one of the main apple production regions in China. Therefore, identifying the genes responsible for drought tolerance and manipulating their expression in genetically modified crops is becoming a critical focus of molecular breeding programmes. To further investigate the function of MdATG18a and explore the initial role of autophagy in apple plants upon drought stress, we generated transgenic tomato and apple plants that overexpressed *MdATG18a* (OE lines) under the control of the CaMV 35S promoter. *MdATG18a* overexpression was associated with enhanced drought tolerance in both species. Under drought stress, when compared with WT, the *MdATG18a* transgenic apple plants showed higher increased expression of *ATG* genes and more autophagosomes. ROS‐scavenging systems were investigated, and we found higher enzymatic activities and more nonenzymatic antioxidants in transgenic apple plants than WT. Activities of CAT and POD, as well as enzymes in ascorbate–glutathione cycle, increased more greatly in transgenic apple plants than WT. The enhanced antioxidative activities and autophagosomes formation might have contributed to less oxidative damage and less accumulations of oxidized proteins observed in the apple transgenics. Thus, *MdATG18a* overexpression enhanced drought tolerance by increasing autophagy, which is thought to be involved in the degradation of oxidized proteins and the regulation of ROS levels under drought stress.

## Results

### Overexpression of *MdATG18a* in tomato enhances drought tolerance

**Figure 1:**
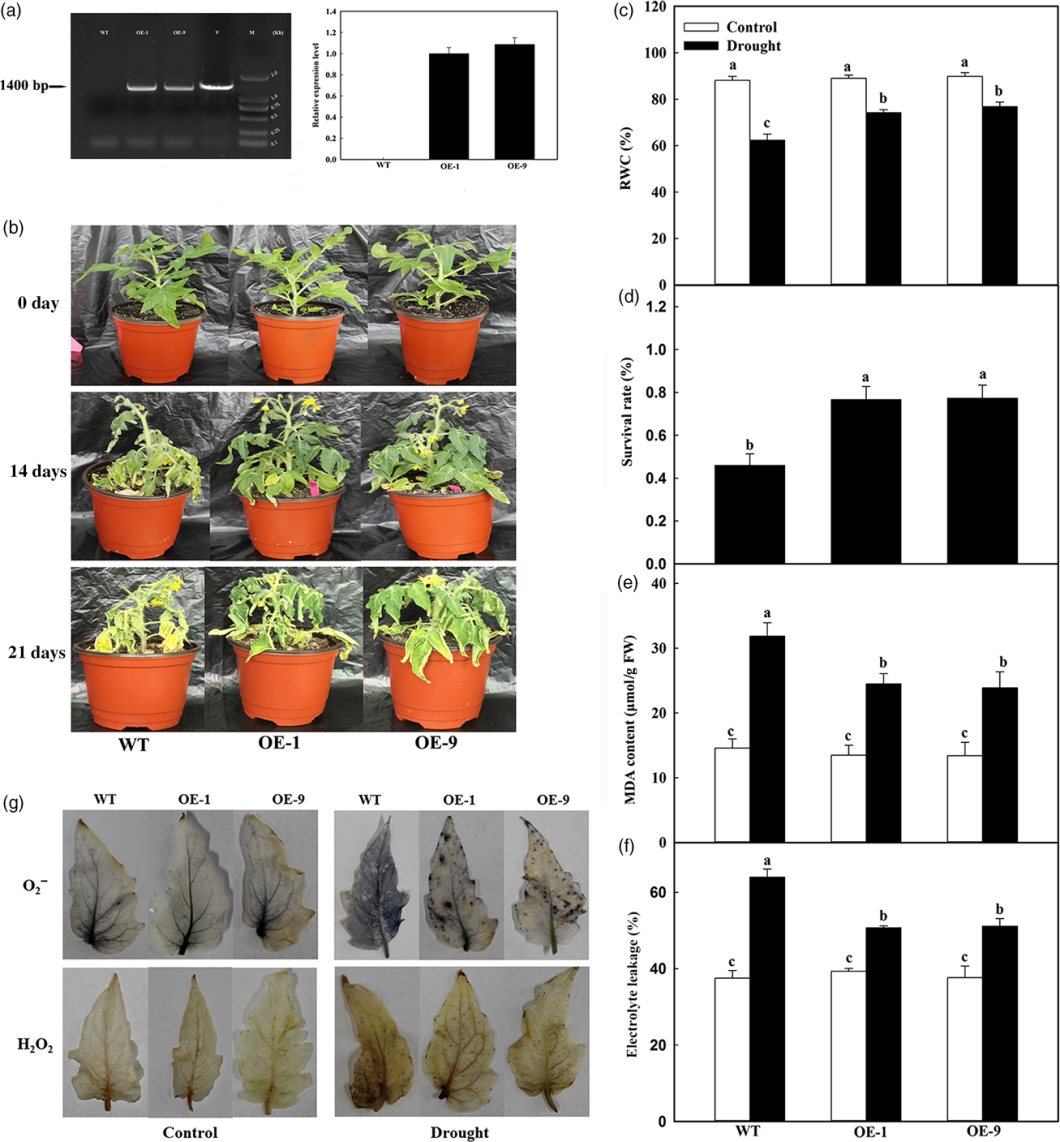
Drought stress tolerance and accumulations of H_2_O_2_ and O_2_^‐^ in *MdATG18a*‐overexpressing tomato. Water was withheld from 3‐week‐old plants for up to 21 days, followed by 3 days of recovery (rewatering). (a) PCR confirmation for transgenic tomato plants. Left panel: PCR with DNA; lanes: M, molecular marker DL2000; V, positive vector containing pCambia2300‐*MdATG18a* plasmid; WT, nontransformed wild type; OE‐1 and OE‐9, *MdATG18a*‐transgenic tomato lines. Right panel: Quantitative RT–PCR analysis of *MdATG18a* expression in leaves of WT and transgenic lines OE‐1 and OE‐9. (b) Increased tolerance in *MdATG18a* OE plants. (c) Comparisons of RWC among WT and OE lines after 21 days of drought treatment. (d) Survival rates of WT and transgenic plants at end of 3‐days recovery period. (e) MDA concentrations in WT and transgenic plants on Day 21 of drought treatment. (f) Electrolyte leakage in WT and transgenic plants on Day 21 of treatment. (g) Results from staining to detect H_2_O_2_ and O_2_^‐^ in leaves from glasshouse‐grown plants exposed to drought treatment for 21 days. Measurements of electrolyte leakage, MDA and RWC were made immediately after the tissues were collected. For H_2_O_2_ and $O_{2}^{-}$, leaves were excised from plants on Day 21 of treatment and immediately placed in NBT for 4 h (H_2_O_2_ test) or DAB for 12 h ($O_{2}^{-}$ test). Data are means of three replicates with SD. Different letters indicate significant differences between treatments, according to one‐way ANOVA Tukey's multiple range tests (*P* < 0.05).

Because *MdATG18a* expression is induced by drought (Wang *et al*., 2014), we overexpressed this gene for further analysis of its biological function under such stress. After its coding region was introduced into a plant‐overexpressing vector under the control of the CaMV35S promoter, we obtained nine transgenic lines (Figure S1a), and two transgenic tomato lines (OE‐1 and OE‐9) had single‐copy insertions (Figure S1b). These two lines constitutively overexpress *MdATG18a* with high mRNA transcript levels (Figure 1a). Under well‐watered conditions, performance did not differ between the WT and the transgenic lines (Figure 1b). However, when drought treatment was applied (i.e. irrigation withheld for 21 days), leaves from WT plants exhibited extensive symptoms of dehydration compared with only slight wilting in OE‐1 and OE‐9 lines after 14 days (Figure 1b). By Day 21, most of the WT leaves were necrotic and kraurotic while those of the transgenic lines remained green and vigorous.

Drought damage was evaluated by measuring relative water content (RWC), electrolyte leakage and levels of malondialdehyde (MDA), which are typical parameters used for assessing tolerance to abiotic stresses in crop plants (Levine *et al*., 1994; Pompelli *et al*., 2010; Tang *et al*., 2013; Wang *et al*., 2012b). Under well‐watered conditions, values for each of these parameters did not differ significantly among genotypes (Figure 1c–e). However, after 14 days of water deprivation, the RWCs of lines OE‐1 and OE‐9 were 19.03% and 23.07% higher, respectively, than the level calculated for the WT (Figure 1c). Survival rates also differed for drought‐stressed plants, that is 76% for the transgenics versus 46% for the WT (Figure 1d). For all plant types, MDA concentrations were increased in response to drought treatment, but the degree of increment was lower in the transgenics (Figure 1e). Finally, our comparison of electrolyte leakage measurements showed that values were 79.2% (OE‐1) and 79.9% (OE‐9) of that determined for the WT (Figure 1f).

The drought‐induced accumulation of highly reactive, toxic ROS can lead to oxidative stress, ultimately damaging various cell components. We used histochemical staining to examine the potential link between *MdATG18a* expression and production of oxide molecules and found that levels of both $O_{2}^{-}$ and H_2_O_2_ were greatly accumulated in response to drought. However, those accumulations were not as high in the OE lines as they were in the WT (Figure 1g).

### Overexpression of *MdATG18a* in apple enhances drought tolerance

**Figure 2:**
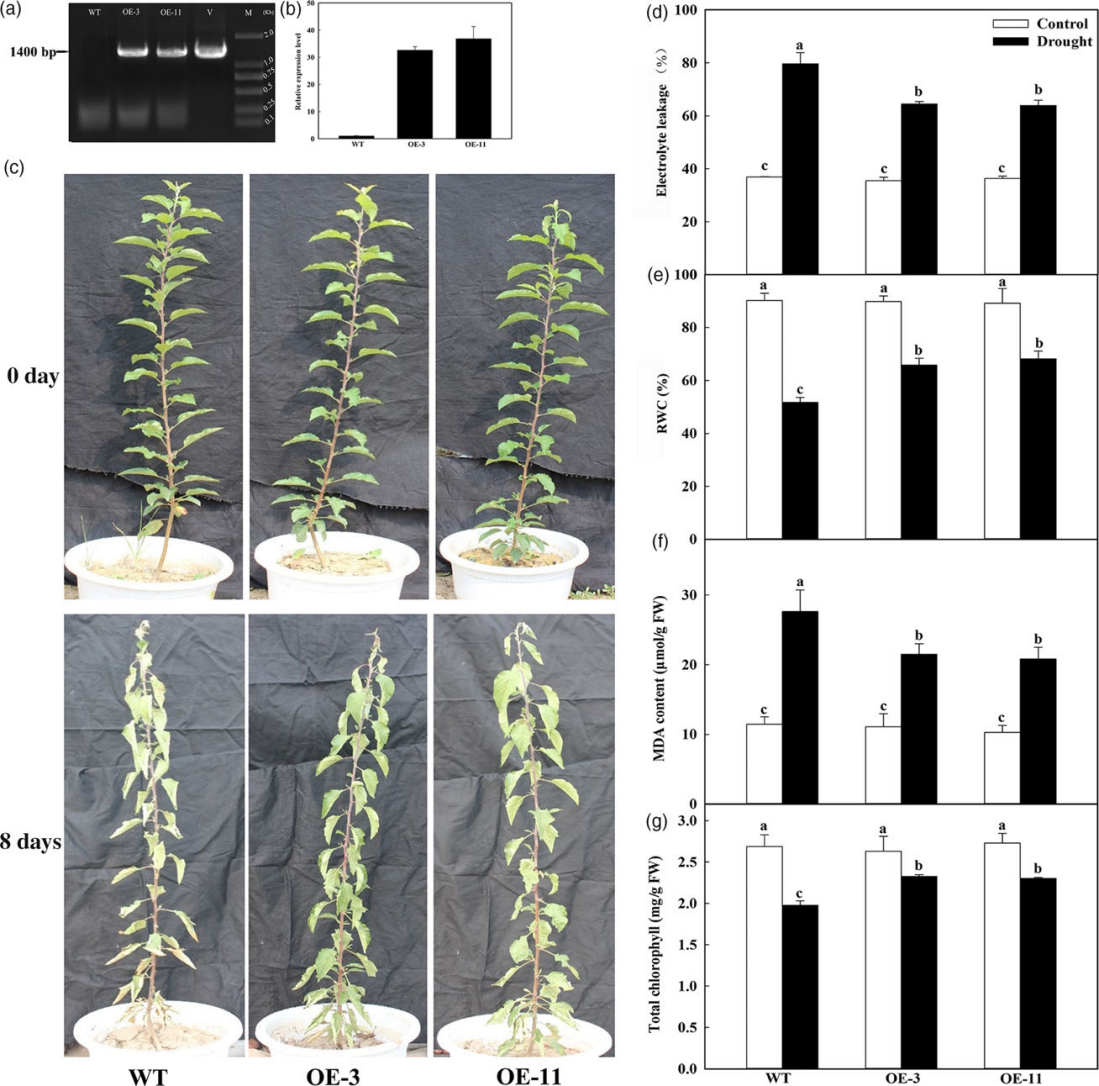
Drought tolerance by *MdATG18a*‐overexpressing apple. Water was withheld from 4‐month‐old plants for up to 8 days. (a) PCR with DNA; lanes: M, molecular marker DL2000; V, positive vector containing pCambia2300‐*MdATG18a* plasmid; WT, nontransformed wild‐type; OE‐3 and ‐11, *MdATG18a*‐transgenic lines. (b) qRT–PCR analysis of *MdATG18a* transcripts in lines OE‐3 and OE‐11; (c) drought tolerance in *MdATG18a* OE plants. (d) Electrolyte leakage in WT and transgenic plants after 8 days of drought stress. (e) Comparisons of RWC from WT and OE lines on Day 8 of treatment. (f) MDA concentrations in WT and transgenic plants on Day 8 of treatment. (g) Total chlorophyll concentrations in WT and transgenic plants on Day 8 of treatment. Data are means of three replicates with SD. Different letters indicate significant differences between treatments, according to one‐way ANOVA Tukey's multiple range tests (*P* < 0.05).

To further investigate the role of *MdATG18a* in drought‐stressed apple, we generated two overexpressing lines with high levels of mRNA transcripts and found that *MdATG18a* transcripts were increased by 32 and 36 times in lines OE‐3 and OE‐11, respectively (Figure 2a,b). Southern blot showed that there were positive hybridization signals in both OE‐3 and OE‐11 plants with single‐copy insertions (Figure S1c).

Under well‐watered control conditions, the phenotypes did not differ significantly between OE lines and the WT. However, after water was withheld for 8 days, the transgenics showed much less wilting and necrosis, and most of their leaves remained vigorous (Figure 2c). Furthermore, their electrolyte leakage was significantly lower than in WT plants on Day 8 (Figure 2d). Although RWCs for all plants were clearly decreased after drought treatment, the rate of that decline was slower in the transgenics, and values were 1.27–1.32 times that of WT plants (Figure 2e). Lower MDA concentrations were detected in the OE lines under drought treatment (Figure 2f). On Day 8 of treatment, total chlorophyll concentrations in OE‐3 and OE‐11 were, respectively, 1.16–1.18 times that of WT plants (Figure 2g). These data demonstrated that overexpression of *MdATG18a* results in less physiological damage to transgenic lines than to the WT under induced drought conditions.

### Apple lines overexpressing *MdATG18a* maintain higher rates of photosynthesis under drought stress

**Figure 3:**
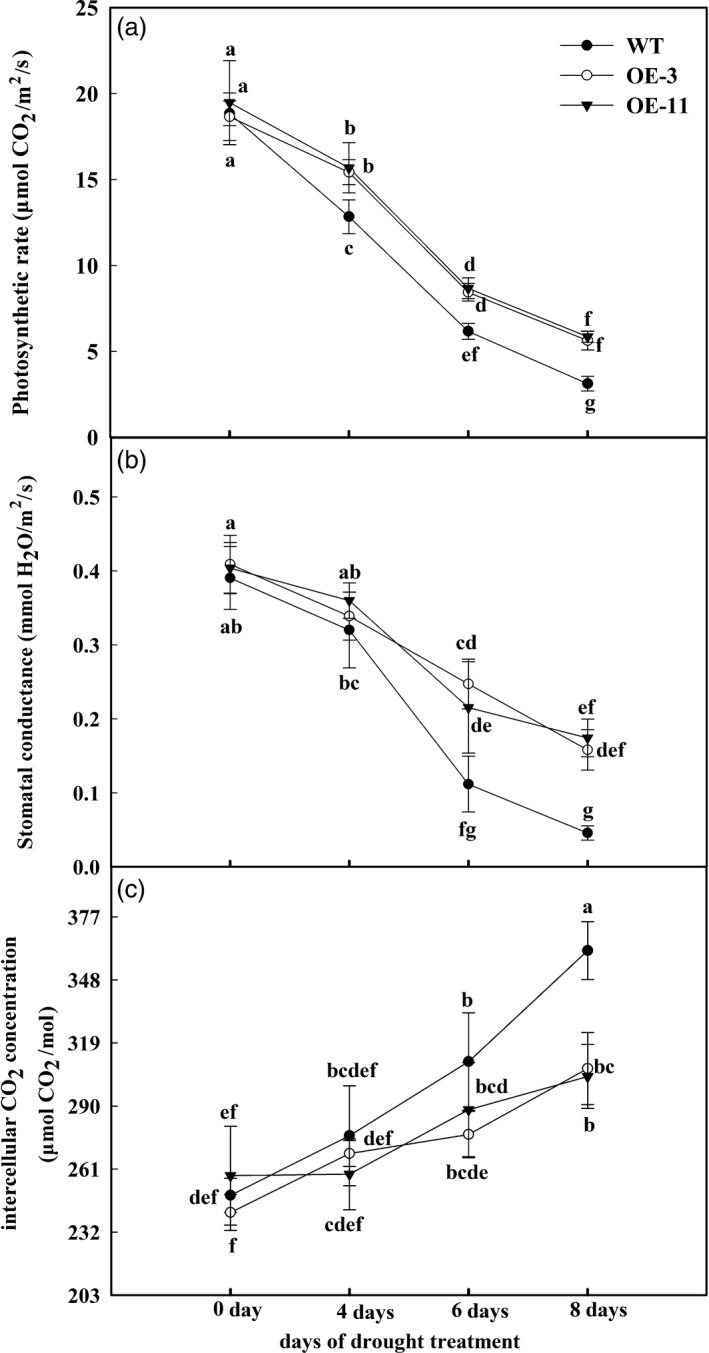
Changes in photosynthesis parameters of *MdATG18a*‐overexpressing apple relative to untransformed plants during period of drought. (a) Photosynthetic rate, (b) stomatal conductance and (c) intercellular CO_2_ concentration. Measurements were made on sunny days between 09:00 and 10:00 h. Data are means of five replicates with SD. Different letters indicate significant differences between treatments, according to one‐way ANOVA Tukey's multiple range tests (*P* < 0.05).

The efficiency of photosynthesis is directly inhibited when stomata close in response to drought stress. To examine the tolerance phenotype of transgenic plants in this respect, we monitored their gas exchange parameters in comparison with performance by the WT. The rate of photosynthesis (Pn), which indicates the assimilation efficiency of CO_2_, was sharply decreased when irrigation was withheld, albeit not to the same extent in the two OE lines as in the WT (Figure 3a). After 8 days of drought treatment, Pn in both OE lines was approximately 1.6 times as high as that in WT plants (Figure 3a). Stomatal conductance followed a similar trend (Figure 3b). Intercellular CO_2_ concentrations gradually increased during the treatment period, but those increments were smaller in the OE lines (Figure 3c). These gas exchange data suggested that plants overexpressing *MdATG18a* maintain a better photosynthetic system under drought conditions.

### Apple lines overexpressing *MdATG18a* accumulate less H_2_O_2_ and show enhanced activities of H_2_O_2_‐scavenging enzymes under drought stress

**Figure 4:**
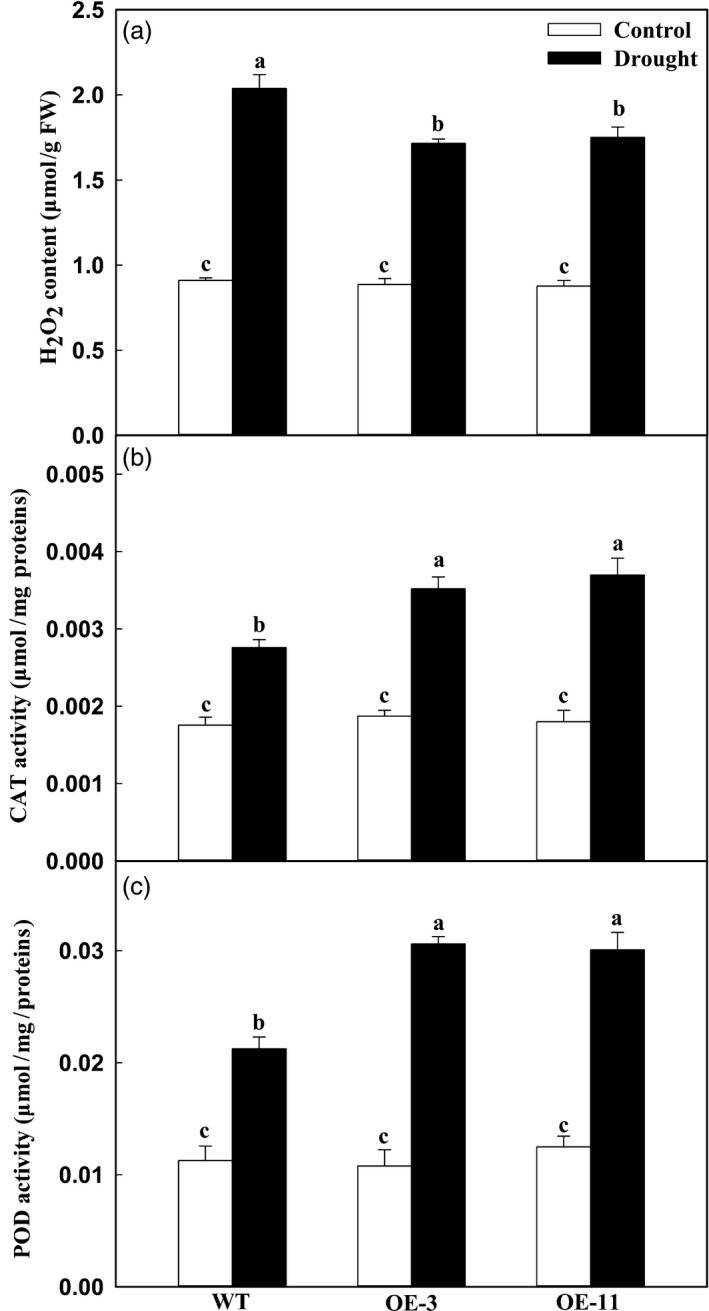
Changes in levels of H_2_O_2_ accumulation and activities of ROS‐scavenging enzymes of apple leaves during drought stress. (a) H_2_O_2_ concentration. (b) CAT activity. (c) POD activity. These data were measured on Day 8 of drought stress. Data are means of three replicates with SD. Different letters indicate significant differences between treatments, according to one‐way ANOVA Tukey's multiple range tests (*P* < 0.05).

As described above, less H_2_O_2_ was accumulated in the *MdATG18a* transgenic tomato plants than in the WT (Figure 1g). To analyse the oxidation status in transgenic apple under drought stress, we measured leaf concentrations of H_2_O_2_ and assayed the activities of major antioxidant enzymes. Although 8 days of drought treatment was associated with great accumulations of H_2_O_2_ in all plant types, significantly lower amounts were detected in the OE lines (Figure 4a). As the main H_2_O_2_‐scavenging enzymes, activities of CAT and POD were obviously increased in response to elevated H_2_O_2_ accumulations. For example, when compared with the well‐watered controls, the increases of CAT activity were 1.88 times for OE‐3 and 2.05 times for OE‐11 versus only 1.57 times in the WT (Figure 4b). A similar pattern was observed for POD activities (Figure 4c). Together, these findings indicated that *MdATG18a* overexpression enhances antioxidant activities in drought‐stressed plants.

### Apple lines overexpressing *MdATG18a* show improved AsA‐GSH cycling under drought stress

**Figure 5:**
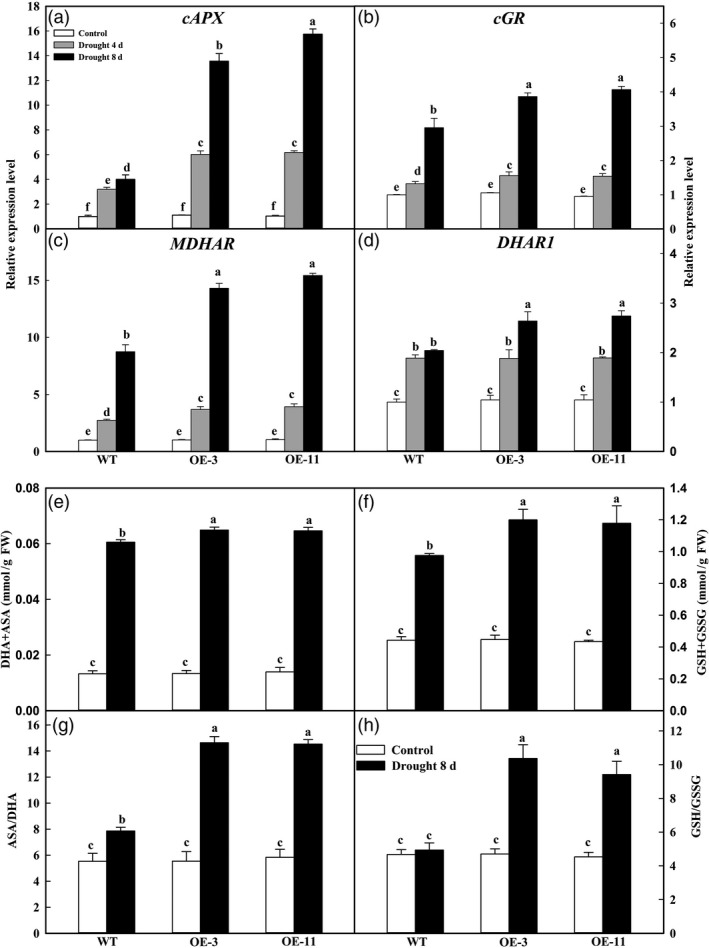
Changes in transcript levels for genes involved in AsA–GSH cycle (a–d) and in concentrations of antioxidants (e–h) of apple leaves during stress period. (a) *APX*, (b) *MDHAR*, (c) *DHAR* and (d) *GR*, (e) ASA+DHA, (f) ASA/DHA, (g) GSH+GSSG, (h) GSH/GSSG. These data were measured on Day 8 of treatment. Expression levels were calculated relative to expression of *Malus* EF‐1α mRNA. Data are means of three replicates with SD. Different letters indicate significant differences between treatments, according to one‐way ANOVA Tukey's multiple range tests (*P *< 0.05).

As an antioxidant system, the AsA–GSH cycle plays an important role in scavenging H_2_O_2_ under stress (Wang *et al*., 2012a). We tested the changes in transcript levels for major genes in that cycle. Under well‐watered conditions, expression of *cAPX*,* MDHAR*,* DHAR1* and *cGR* did not differ significantly among genotypes (Figure 5a–d). However, as the drought period became prolonged, transcript levels gradually increased, especially in the transgenic lines. For example, on Day 8 of treatment, expression of *cAPX* was 3.38 and 3.93 times that of the WT in OE‐3 and OE‐11, respectively (Figure 5a).

We further examined the regulation of *MdATG18a* overexpression in AsA‐GSH cycle by evaluating the states of ascorbate and glutathione. No significant changes in levels of total ascorbate (reduced ascorbate AsA + dehydroascorbate DHA) and total glutathione (reduced glutathione GSH + oxidized glutathione GSSG) were found between the OE lines and WT plants under well‐watered conditions (Figure 5e–h). However, after 8 days of drought treatment, levels of total ascorbates were significantly higher in the transgenics (Figure 5e). This difference was more remarkable in terms of the ratio of AsA to DHA, which was 1.86 and 1.85 times that of the WT in lines OE‐3 and OE‐11, respectively (Figure 5g). Similar results were determined for the total glutathione pool and the GSH/GSSG ratio (Figure 5f,h). Upon induction of drought stress, that ratio did not change in the WT, but increased to 2.11 times for OE‐3 and to 1.75 times for OE‐11 (Figure 5h). Changes in the levels and status of AsA and GSH were in accord with the patterns of transcriptional expression for the main enzymes involved in AsA‐GSH recycling. This clearly demonstrated that overexpressing plants have greater capacity for recycling as well as for maintaining higher amounts of antioxidants.

### Apple lines overexpressing *MdATG18a* accumulate smaller amounts of insoluble and oxidized proteins under drought stress

**Figure 6:**
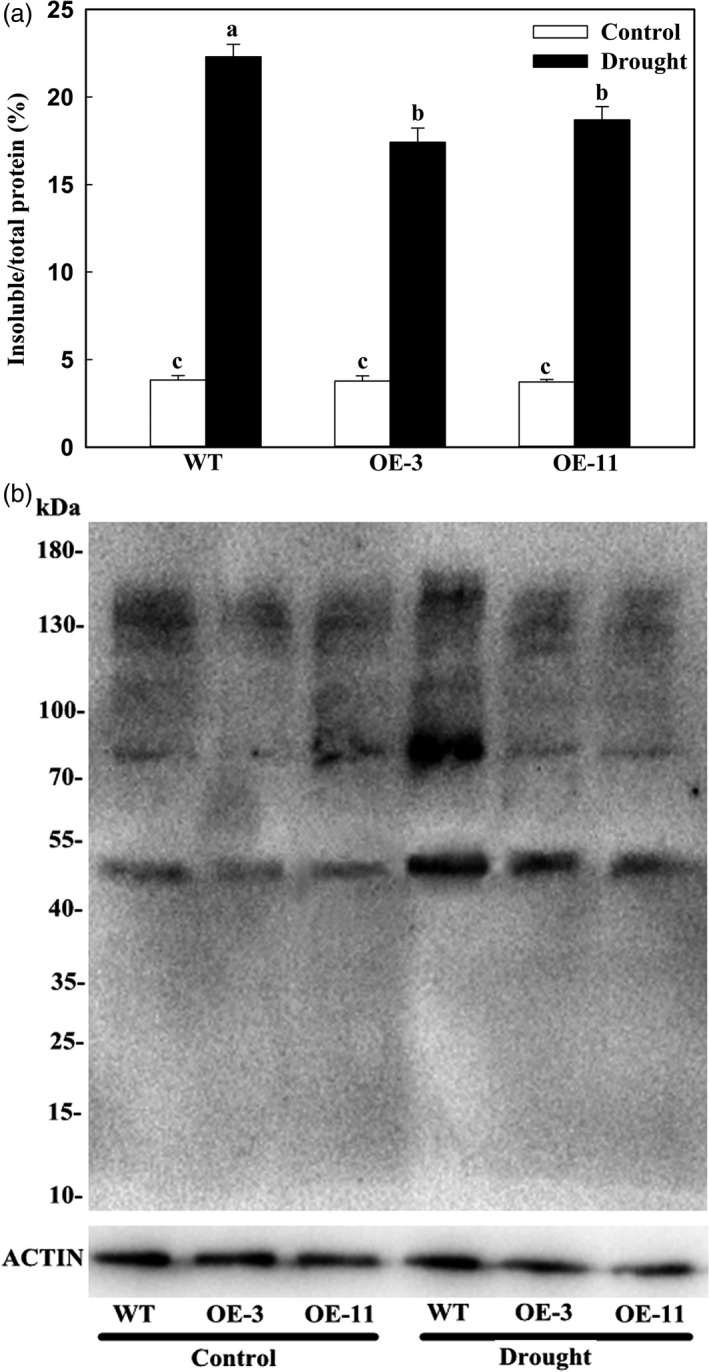
Accumulation of insoluble proteins and oxidation of soluble proteins in apple leaves on Day 8 of drought stress. (a) Accumulation of insoluble proteins in WT and transgenic plants on Day 8 of drought stress. Percentages of insoluble proteins to total proteins were calculated based on amount of total proteins in homogenates at beginning of period and insoluble proteins in final pellets. Data are means of three replicates with SDs. Different letters indicate significant differences between treatments, according to one‐way ANOVA Tukey's multiple range tests (*P* < 0.05). (b) Oxidation of soluble proteins. Leaf samples were collected after 8 days of treatment, and soluble proteins were isolated and derivatized by 2,4‐dinitrophenol (DNP), followed by immunoblotting with an anti‐DNP antibody. Molecular size markers are indicated at left. Amount of protein extract‐loading was referenced by immunoblot analysis with antiactin antibody (lower panel).

Autophagy plays a key role in the degradation of oxidized proteins and damaged organelles when plants are stressed (Xiong *et al*., 2007b). To analyse the relationship between *MdATG18a* expression and the capacity for such degradation, we measured levels of insoluble and oxidized proteins. In the absence of drought conditions, the amount of insoluble protein as a percentage of the total did not differ between the OE lines and the WT (Figure 6a). However, after 8 days of treatment, insoluble proteins were greatly accumulated in all plants, but those levels were significantly lower in the transgenics. Meanwhile, the proportion of oxidized proteins was also increased by drought in all plants, but accumulations were lower in the OE lines (Figure 6b). These findings strongly suggested that overexpression of *MdATG18a* can cause oxidized proteins to be degraded and the level of insoluble aggregates to decline when plants are exposed to drought conditions.

### Apple lines overexpressing *MdATG18a* show up‐regulated expressions of other *MdATG*s and increased formation of autophagosomes under drought stress

**Figure 7:**
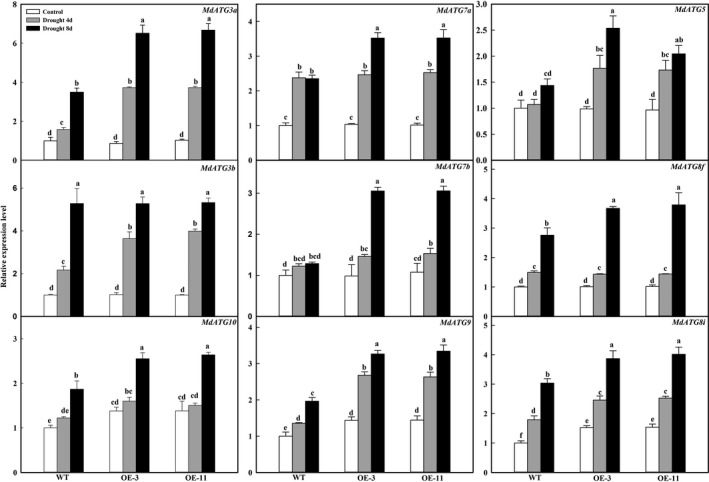
Changes in transcription level of apple autophagy‐related genes during drought period. Total RNA was isolated from leaf samples collected at indicated times, and expression levels were calculated relative to expression of *Malus EF‐1*α mRNA. Data are means of three replicates with SD. Different letters indicate significant differences between treatments, according to one‐way ANOVA Tukey's multiple range tests (*P* < 0.05).

To investigate the occurrence of autophagy in response to drought stress, we examined the expression patterns of several important *ATG* genes and compared the numbers of autophagosomes that formed in the transgenic lines and WT plants. Expression of *MdATG8i*,* MdATG9* and *MdATG10* was significantly higher in OE plants than in the WT, even under well‐watered control conditions (Figure 7). On days 4 and 8 of drought treatment, those three genes, as well as *MdATG3a*,* MdATG5*,* MdATG7a*,* MdATG7b*,* MdATG8c* and *MdATG8f*, were up‐regulated in all genotypes but transcript levels were significantly greater in the OE lines than in the WT.

**Figure 8:**
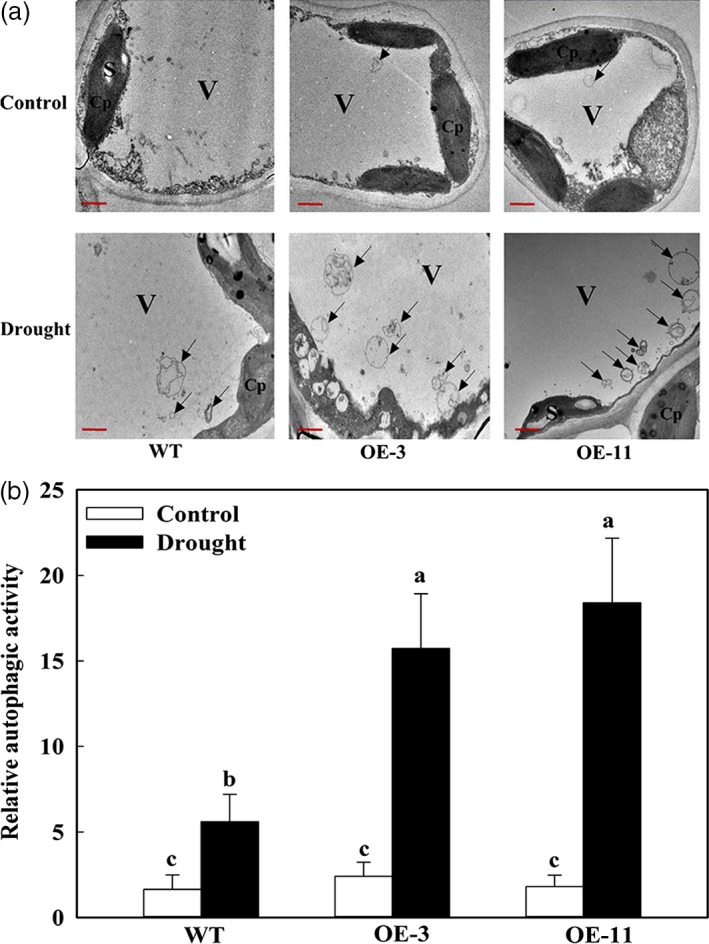
Visualizing the accumulation of autophagosomes in apple leaves under drought stress. (a) Representative TEM images of autophagic structures in mesophyll cells from WT and *MdATG18a* OE plants. V, vacuole; S, starch; Cp, chloroplast. Autophagic bodies are indicated by black arrows. Bars: 1 μm. (b) Relative autophagic activity normalized to activity of WT or *MdATG18a* OE plants shown in (a). More than 10 cells were used to quantify structures. Data are means of three replicates with SD. Different letters indicate significant differences between treatments, according to one‐way ANOVA Tukey's multiple range tests (*P* < 0.05).

We used transmission electron microscopy (TEM) to observe autophagosome formation in response to drought and found very few autophagosome structures in the leaves under well‐watered conditions, regardless of plant type (Figure 8a). However, after 6 days of treatment, up to three times as many autophagosomes and autophagic bodies had accumulated in the OE lines than in WT plants (Figure 8a,b). This demonstrated that the occurrence of autophagy in apple is significantly enhanced by overexpression of *MdATG18a* when plants are challenged by drought stress.

## Discussion

Autophagy is a conserved cellular degradation process in eukaryotes for survival during environmental stresses and for cellular remodelling during development (Lai *et al*., 2011; Xiong *et al*., 2007b). We previously cloned *MdATG18a* gene from *Malus domestica* (Wang *et al*., 2014). Sequence analyses indicated that MdATG18a has typical WD40‐repeat domains which usually serve as scaffolds or platforms for protein–protein interactions and the assembly of protein complexes (Smith *et al*., 1999). WD40‐repeat proteins are relatively abundant in eukaryotes and are implicated in various important functions including signalling transduction, transcriptional regulation, cell cycle control, apoptosis and autophagy (Nair *et al*., 2010). *MdATG18a* showed obvious up‐regulation under leaf senescence and drought stress (Wang *et al*., 2014). Herein, we used *MdATG18a* overexpressing plants of tomato and apple to gain more insight into its function and mechanism associated with improved drought tolerance. Our overall data clearly demonstrated that these transgenic plants have improved tolerant to drought stress. Upon drought stress compared with WT, the apple transgenic plants have less damage indicated by parameters of RWC, MDA levels, ROS levels and electrolyte leakage and maintain higher chlorophyll contents and photosynthesis rates, as well as antioxidant ability, which is evidenced by increased activities of H_2_O_2_‐scavenging enzymes CAT and POD and greater capacity for AsA‐GSH recycling. Most importantly, there were less oxidized proteins aggregated in *MdATG18a* OE apple lines, which might be explained by the up‐regulated expressions of other *MdATGs* and increased formation of autophagosomes under drought stress.

When plants are subjected to drought conditions, RWC values tend to decline and are used as an important indicator of the capacity for water retention (Wang *et al*., 2012b). We found here that RWC was significantly higher in the transgenic tomato and apple plants than in the WT. Changes in MDA levels and electrolyte leakage in response to drought are two other efficient indicators when evaluating membrane integrity and the extent of tolerance to abiotic stress (Liao *et al*., 2016; Tang *et al*., 2013). Here, MDA concentrations and electrolyte leakage were significantly higher in WT tomato and apple plants than in their corresponding transgenics when exposed to drought conditions. This demonstrated that cell damage is alleviated by the overexpression of *MdATG18a*. The onset of abiotic stress leads to increased production of ROS, causing oxidative damage to cellular components (Kasukabe *et al*., 2004). Our transgenic tomato plants accumulated less H_2_O_2_ and $O_{2}^{-}$ than did the WT after 21 days of treatment. We noted a similar trend in the detection of H_2_O_2_ in transgenic apple lines.

Plants utilize both enzymatic and nonenzymatic mechanisms for scavenging ROS (Cheng *et al*., 2016; Shigeoka *et al*., 2002; Wang *et al*., 2015a; Zhou *et al*., 2013). For example, under drought treatment, overexpression of *VqbZIP39* in *Arabidopsis* increases the activities of CAT, POD and SOD (Tu *et al*., 2016). Overexpression of *MdcyMDH* also enhances SOD and CAT activities in transgenic apple callus and in transformed tomato plants (Yao *et al*., 2011). Likewise, we found that activities of CAT and POD were significantly higher in transgenic apple than in the WT under drought stress. As the most important scavenger of H_2_O_2_ in green tissues (Shigeoka *et al*., 2002), APX is located in different cellular compartments and works in conjunction with the AsA–GSH cycle. Overexpression of *MdATG18a* up‐regulated the expression of *cAPX*, as well as *MDHAR*,* DHAR* and *cGR* in that cycling system (Figure 5a–d). The trend in AsA and GSH concentrations and interconversions between their reduced and oxidized forms also fit with the pattern of transcriptional changes we noted in cycling. The levels of total AsA and total GSH, as well as the ratios of AsA/DHA and GSH/GSSG, were significantly elevated in *MdATG18a* transgenic apple under drought stress. Strong maintenance of a reduced AsA state might be due to the high expression levels of *MDHAR* and *DHAR*, which are involved in AsA recycling (Conklin and Barth, 2004). This drought tolerance improvement by *MdATG18a* overexpression is in accordance with a recent report about transgenic apple plants overexpressing *MdcyMDH*, which show greater tolerance to salt and cold stresses because the redox state is modified by increases in AsA and GSH levels as well as a reduction in their oxidized ratios (Wang *et al*., 2016). Drought‐related damage is presumably modulated in *MdATG18a* transgenic plants because of improvements in their antioxidant systems.

As one of the key processes in primary metabolism, photosynthesis can be affected when water deficits lead to stomatal closure (Chaves *et al*., 2009). Abiotic stress‐generated ROS can damage the photosynthetic apparatus and inhibit PSII repair because of an imbalance in the redox system in chloroplasts (Gururani *et al*., 2015). Intracellular ROS accumulation and its inhibition on photosynthesis can be reduced by engineering the production of ROS‐scavenging enzymes such as CAT and APX and by increasing the levels of antioxidants such as AsA and GSH (Gururani *et al*., 2015). The enhancement of Pn that we found in transgenic apple plants might be a result of an improved antioxidant system under drought stress. Autophagy has a critical role in chloroplast degradation (Wada *et al*., 2008) and might constitute a dedicated and dynamic quality control mechanism of chloroplasts. However, little is known about how autophagy directly regulates the photosynthetic apparatus or helps in repairing important proteins such as D1 protein.

Autophagy is thought to be involved in degrading oxidized proteins and regulating ROS levels under osmotic or salt stress (Xiong *et al*., 2007b). It can remove misfolded and damaged proteins or protein aggregates as a mechanism for protein quality control under various stress conditions, such as heat (Yang *et al*., 2016; Zhou *et al*., 2014a). *Arabidopsis atg5*,* atg7* and *nbr1* mutants have compromised heat tolerance because the plants accumulate insoluble and highly ubiquitinated proteins (Zhou *et al*., 2014a). Heat stress induces the expression of ATG genes and the accumulation of autophagosomes in tomato plants. Their tolerance to such stress is thought to be mediated by cooperative regulation by both WRKY33 and ATG proteins, probably through the removal of heat‐induced protein aggregates (Zhou *et al*., 2014a). In tomato, *HsfA1a* confers drought tolerance through autophagy activation, by which ubiquitinated protein aggregates are degraded in response to stress (Wang *et al*., 2015b). We also found that insoluble and oxidized proteins were accumulated under drought conditions. However, overexpression of *MdATG18a* in apple reduced those stress‐related accumulations. The lower amounts of oxidized proteins measured in our OE lines might be explained by activation of autophagy via *MdATG18a* overexpression. This is supported by the fact that major *MdATG* genes were significantly up‐regulated and autophagosomes formed in large numbers in the transgenic plants.

Use of TEM is a valid and important method for the quantitative analysis of autophagosomes (Cheng *et al*., 2016; Klionsky *et al*., 2012). Our transgenic lines had more autophagosomes than WT under drought stress, a finding that was in line with the decreased accumulation of oxidized insoluble proteins under drought stress. These data again suggest that *MdATG18a* plays a positive role in removing misfolded and oxidized proteins in drought‐stressed apple, probably through the activation of autophagy.

In conclusion, we have functionally characterized *MdATG18a* by overexpressing it in tomato and apple. Transgenic apple plants had enhanced drought tolerance, possibly because of a more‐reductive redox state. We propose that active autophagy, as demonstrated by the up‐regulation of autophagy‐related genes and greater autophagosome accumulations in transgenic plants, might contribute to better quality control of proteins and a balanced antioxidant environment under drought stress. Our discoveries provide an interesting link between autophagy and ROS‐scavenging systems, and they show that MdATG18a functions positively in apple drought tolerance. This is a promising perspective for future efforts in crop breeding.

## Experimental procedures

### Plant materials and treatments

Seeds of *Solanum lycopersicum* cv. ‘Micro‐Tom’ from transgenic and WT plants were germinated in 250‐cm^3^ plastic pots in a controlled environment walk‐in chamber. Growing conditions for these tomato plants included 25 °C, 140 μmol photons per m^2^ per s, 70% relative humidity and a long‐day (14‐h) photoperiod. The seedlings were watered regularly and supplied with half‐strength Hoagland's nutrient solution (pH 6.0) once a week for 20 days to maintain healthy growth. To induce drought stress, some were then exposed to water deprivation by withholding water by 21 days. After drought treatment, the seedlings were rehydrated for 4 days to recover, and those plants with upper leaves turning alive were considered as survived (Zhu *et al*., 2015). As the well‐watered control group, other plants continued to receive normal irrigation. Samples were harvested on days 0, 14 and 21 of this experimental period, collecting three biological replicates per treatment.

Tissue‐cultured plants of *Malus domestica* cv. ‘Roya Gala’ were initially grown on an MS agar medium containing 0.3 mg/L 6‐BA and 0.2 mg/L IAA. They were cultured under conditions of 23 °C, 60 μmol/m^2^/s and a 14‐h photoperiod. After rooting on MS agar media containing 0.5 mg/L IBA and 0.5 mg/L IAA, the transgenic and WT plantlets were transferred to small plastic pots (8.5 × 8.5 × 7.5 cm) containing a mixture of soil/perlite (1 : 1, v : v). After one month of adaptation in a growth chamber, the plants were moved to large plastic pots (30 × 26 × 22 cm) filled with a mixture of forest soil/sand/organic substrate (5 :1 : 1, v : v : v) and grown in the glasshouse. They were watered regularly and supplied with half‐strength Hoagland's nutrient solution (pH 6.0) once a week. After three months of growth under these conditions, healthy and uniformly sized plants were assigned to two treatment groups. Half were subjected to drought stress by withholding water, while the other (well‐watered control) continued to receive daily irrigation so that a saturated soil water content was maintained. On days 0, 4, 6 and 8 of this experiment, between 10:00 and 11:00 h, the ninth to twelfth leaves from the base of a stem (fully mature leaves) were sampled from five trees per treatment. They were pooled together and divided into three repeats. After being rapidly frozen in liquid nitrogen, they were stored at −80 °C.

### Construction of plant‐overexpressing vector for *MdATG18a*

The coding region of *MdATG18a* was introduced into the pCambia2300 vector by *Xba*I and *Kpn*I. Primers with restriction sites are listed in Table 1. This vector was driven by the CaMV 35S promoter and carried the kanamycin (Kan) selectable marker in plants. Sequencing‐confirmed plasmid was transformed to *Agrobacterium* EHA105 by electroporation (Hood *et al*., 1984).

**Table 1:** Primers used in this study

Name/Accession no.	Sequence (5′‐3′)	Purpose
oe*ATG18a*	F: GCTCTAGAATGGCCACCCTCTCCGC	Vector construction for plant transformation
	R:GGGGTACCTTAAAAGGCTTCTTCGGGCTTTA	
q*ATG18a/* KC800804	F: ATGATTCCAGGCTTGCCTGCTTTG	Quantitative expression of *MdATG18a*
	R: TGCAGCAAAGTTCCGTCGAGAGTA	
*EF‐1*α*/* DQ341381	F: ATTCAAGTATGCCTGGGTGC	Real‐time PCR using *Malus EF‐1*α as reference gene
	R: CAGTCAGCCTGTGATGTTCC	
d*ATG18a*	F: GAGAACACGGGGGACTCTAGA	DNA confirmation of *MdATG18a* for transgenic identification
	R: CGATCGGGGAAATTCGAGCTC	
q*cAPX/* EF528482	F: AACTACAAGGGATGAAGCC	Quantitative expression of *MdcAPX*
	R: CAACGAGGATGATAACCAG	
q*MDHAR/* FJ752239	F: CCATACTTCTATTCCCGCTCCT	Quantitative expression of *MdMDHAR*
	R: CGACCACCTTCCCGTCTTT	
q*DHAR1/* DQ322706	F: AGTGGACGGTTCCAGCAGA	Quantitative expression of *MdDHAR1*
	R: AGTGGACGGTTCCAGCAGA	
q*GR/* JF268781	F: GTTCAGCGACAAGGCGTAT	Quantitative expression of *MdGR*
	R: TCAACCGATTTCCATTTCC	
q*ATG3a/* KF438032	F: AAGGGGGCGGAGATGGTTC	Quantitative expression of *MdATG3a*
	R: GCACTTAGAGACGAGGTTATCGC	
*qATG3b/* KR024682	F: AGGGAGATGGTTTTGAAACAGA	Quantitative expression of *MdATG3b*
	R: ACTTAGAGACGAGGTTATCGC	
q*ATG5/* *KY305671*	F: GCAGGTCGTGTTCCAGTTC	Quantitative expression of *MdATG5*
	R: CCTCCTCCTCCTTGTATCTCAA	
q*ATG7a/* KF438034	F: GCGGATATGAGCAACCTTGGC	Quantitative expression of *MdATG7a*
	R: ATCAATAGGCGCAACGACATCA	
q*ATG7b/* KF438035	F: ATCGGTAACAGGAGTAAGTCGG	Quantitative expression of *MdATG7b*
	R: TTTATCAAGCGCATGAAAGCCT	
q*ATG8f/* *KF438036*	F: TCGTAGACAATGTCCTCCCAGC	Quantitative expression of *MdATG8f*
	R: CCAAATGTGTTCTCGCCACTGT	
q*ATG8i/* KF438037	F: GCAGCAGGCTTCACTTGACTCC	Quantitative expression of *MdATG8i*
	R: GGAATCCATGCGACTGGCTGTT	
q*ATG9/* KF438038	F: ACTTCATGCGTCAGCCTTCAGA	Quantitative expression of *MdATG9*
	R: CGTTCCTCCAATCCAACCGTTG	
q*ATG10/* KF438033	F: TGGAACCAGCGAGTGGATGAAG	Quantitative expression of *MdATG10*
	R: ACAACTGAGAGCCAAGACACCA	
*Actin/*Sl11g005330	F: TGTCCCTATTTACGAGGGTTATGC	Real‐time PCR of *actin* as a reference gene in tomato
	R: CAGTTAAATCACGACCAGCAAGAT	

### Genetic transformation of tomato and apple

‘Micro‐Tom’ tomato was transformed via *Agrobacterium*‐mediated method, as previously described (Guo *et al*., 2012). The resultant plants were PCR‐confirmed to select positive transgenic lines. Nine transgenic lines were obtained at the beginning, and they were kept screening with 100 mg/L Kan. OE‐1 and OE‐9 were two lines that did not segregate anymore until F2 generation. Meanwhile, Southern blot checked the copy numbers of these lines and confirmed the single‐copy insertions in OE‐1 and OE‐9 lines. Therefore, these two homozygous lines were selected for further tests.

Transgenic ‘Royal Gala’ apple plants were generated from leaf fragments through *Agrobacterium*‐mediated transformation, as previously described (Dai *et al*., 2013). Regenerated Kan‐resistant buds were subcultured every three weeks, based on selection with 25 mg/L Kan. Lines that stopped growing or died were eliminated and only those showing normal growth were maintained for further verification. We performed PCR analysis with isolated DNA to check for the presence of the transgene in putative transformed lines. Plasmid and nontransformed plant DNA were used as the positive and negative controls, respectively. Total RNA was then isolated from positive lines as well as from the nontransformed WT according to a CTAB method (Chang *et al*., 1993). Overexpression of *MdATG18a* was confirmed by quantitative real‐time PCR (qRT–PCR).

### Southern blot

The PCR‐positive transgenic tomato and apple lines were analysed for insertions by Southern blotting. Primers (forward: AGATCCTCGCCGTCGGGCATG and reverse: AGATCCTCGCCGTCGGGCATG) were used to amplify the *NPTII* fragment to prepare the labelled probe using the PCR DIG Probe Synthesis Kit (Roche, Mannheim, Switzerland). Genomic DNA was extracted from fresh young leaves of every transgenic tomato and apple line and nontransgenic controls. Approximately 40 μg of genomic DNA from each genotype was used and completely digested with the restriction enzyme *EcoR*I, and the plasmid was digested with the restriction enzyme *Xba*I. Southern blotting was performed according to the DIG DNA Labeling and Detection Kit (Roche, Switzerland) manual instructions.

### RNA extraction, DNA isolation and qRT–PCR

Total RNA was extracted according to a CTAB method (Chang *et al*., 1993). The DNA was removed by treating with RNase‐free DNase I (Thermo Scientific, Waltham, MA). Apple genomic DNA was extracted by a modified CTAB method (Modgil *et al*., 2005), and first‐strand cDNA was synthesized using a RevertAid First Strand cDNA Synthesis Kit (Thermo Scientific) with the same amount of mRNA (1 μg). qRT–PCR was performed with the iQ5 Multicolor Real‐Time PCR Detection System (Bio‐Rad Laboratories, Hercules, CA) and SYBR Green Master Mix (Takara, Dalian, China). Transcripts of the *Malus* elongation factor 1 *alpha* gene (*EF‐1*α; DQ341381) were used to standardize the cDNA samples for different genes. Specific primer sequences for expression analysis are shown in Table 1. All experiments were repeated three times biologically, based on three separate RNA extracts from three repeats.

### Measurements of gas exchange parameters

Gas exchange parameters were monitored by a LI‐COR 6400 portable photosynthesis system (LI‐COR, Huntington Beach, CA). Measurements were performed on the ninth to twelfth leaves from the base of selected plant stems on sunny days between 09:00 and 10:00 h. All photosynthetic measurements were recorded at a constant airflow rate of 500 μmol/s. The concentration of CO_2_ was 400 ± 5 cm^3^/m^3^, and the temperature was 28 ± 2 °C. The rate of photosynthesis (Pn), intercellular CO_2_ concentration and stomatal conductance were obtained from five plants per treatment. Measurements were made at a photosynthetic photon flux density of 1000 μmol/m^2^/s, as provided by a Q‐Beam (blue and red diode) light source.

### Evaluation of stress tolerance

Relative water contents were determined as previously described (Gaxiola *et al*., 2001). Briefly, fresh weights (FWs) of the terminal leaflets were measured before the leaves were immersed in distilled water under darkness for 24 h to obtain their fully turgid weights (TWs). They were then oven‐dried at 70 °C for 72 h before their dry weights (DWs) were recorded. The relative water content was calculated as follows:

$$\text{RWC}\;\left(\%\right)=\frac{\text{FW}-\text{DW}}{\text{TW}-\text{DW}}\times 100.$$

Chlorophyll was extracted with 80% acetone, and concentrations were determined spectrophotometrically according to the method of Lichtenthaler and Wellburn (1983). Levels of MDA were obtained as previously described (Heath and Packer, 1968). Electrolyte leakage in the leaves was calculated according to an earlier method (Thalhammer *et al*., 2014). Finally, H_2_O_2_ was extracted with 5% (w/v) trichloroacetic acid and measured as described previously (Patterson *et al*., 1984).

### Detection of H_2_O_2_ and superoxide ion ($O_{2}^{-}$)

Accumulations of $O_{2}^{-}$ and H_2_O_2_ were examined by histochemical staining methods that used nitro blue tetrazolium (NBT) and diaminobenzidine (DAB), respectively. For $O_{2}^{-}$ detection, leaves were incubated under darkness for 4 h at 25 °C in fresh NBT solution (1 mg/mL) prepared in 10 mm Hepes (pH 7.5) with 0.1% Triton X‐100. For H_2_O_2_ detection, leaves were placed in a fresh DAB (1 mg/mL) solution prepared in 10 mm sodium phosphate buffer (pH 7.5) with 0.1% Triton X‐100. The samples were then incubated in a growth chamber overnight until brown spots were visible. The chlorophyll was removed by immersing the leaves in 90% ethanol and heating in a boiling water‐bath for 15 min. Samples were fixed in 90% ethanol with 20% glycerol at 4 °C prior to photographing (Rangani *et al*., 2016; Wang *et al*., 2015a).

### Extraction and assay of antioxidant enzymes

Apple leaves (0.1 g) were ground with a 1.2 mL ice‐cold buffer containing 50 mm potassium phosphate buffer (pH 7.8), 1 mm EDTA‐Na_2_, 0.3% Triton X‐100 and 1% (w/v) polyvinylpolypyrrolidone. The homogenate was centrifuged at 13 000 ***g*** for 20 min at 4 °C, and the supernatant was used for the assays. Activities of CAT and POD were determined according to established protocols (Wang *et al*., 2012a; Zhang *et al*., 2015).

### Extraction and analysis of antioxidant metabolites

Both AsA and DHA were extracted with 6% (v/v) HClO_4_, while GSH and GSSG were extracted with 5% (v/v) sulfosalicylic acid. Their concentrations were measured as previously described (Wang *et al*., 2012a).

### Detection of insoluble proteins and Western blotting

Soluble, insoluble and total proteins were measured according to earlier methods (Zhou *et al*., 2013). Their concentrations were determined with protein assay kits (Bio‐Rad), using bovine serum albumin as a standard. Oxidized proteins from the soluble protein fraction were detected with an OxyBlot protein oxidation detection kit (Chemicon International, Temecula, CA), based on the manufacturer's instructions. Actin was monitored with a monoclonal antibody (CWBIO, Beijing, China). After incubation with a horseradish peroxidase‐linked secondary antibody (CWBIO), the antigen–antibody complexes were detected using Clarity™ Western ECL Substrate (Bio‐Rad) according to the manufacturer's instructions.

### Transmission electron microscopy analysis

Our TEM analysis was performed as previously described, but with slight modifications (Wang *et al*., 2015b). On Day 6 of the drought stress period, mature leaves were excised from the apple plants and immediately cut into small pieces and then fixed with 2.5% glutaraldehyde in 0.2 m PBS buffer (pH 7.4) before being placed under darkness for 12 h at 4 °C. After washes with PBS buffer, the samples were fixed for 2.5 h in 1% (v/v) osmium tetroxide at room temperature. They were then dehydrated in a graded ethanol series (30%–100%; v/v) and embedded in Epon 812. Ultrathin sections (70 nm) were prepared on an ultramicrotome (Leica ULTRACUT, Wetzlar, Germany) and collected on Formvar‐coated grids. The sections were examined using a JEOL‐1230 transmission electron microscope (Hitachi, Tokyo, Japan) at an accelerating voltage of 80 kV to observe autophagosomes and autophagic bodies.

### Statistical analysis

Three independent replicates were used for each determination. Experimental data were presented as means ± standard deviation (SD). Statistical data analysis was performed via one‐way ANOVA, followed by Tukey's multiple range tests, using the SPSS 18 statistical package SPSS, Chicago, Illinois, USA. Differences among results were considered statistically significant at *P* < 0.05.

## Conflict of interest

The authors declare no conflicts of interest.

## Supporting information


**Figure S1** Southern blot of *MdATG18a* transgenic lines of tomato and apple.
