# Supplementary material for: Improvement of drought tolerance by overexpressing MdATG18a is mediated by modified antioxidant system and activated autophagy in transgenic apple
Source: Plant Biotechnol J. 2017 Aug 22;16(2):545–57. doi: 10.1111/pbi.12794 (PMC5787838; doi:10.1111/pbi.12794)
Supplement: Supplementary file 1 — Figure S1 Southern blot of MdATG18a transgenic lines of tomato and apple. [file PBI-16-545-s001.pdf]

Supplemental data

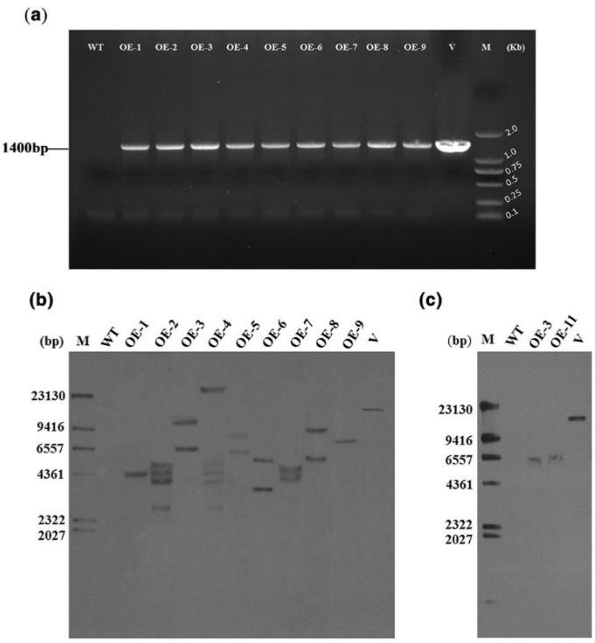

Fig. S1 Southern blot of *MdATG18a* transgenic lines of tomato and apple. Young tomato and apple leaves samples were collected at normal growth condition. (a) PCR with DNA of nine tomato transgenic lines. Lanes: WT, wild type ‘Micro-Tom’ plants; OE-1~9, nine transgenic lines; V, positive vector containing pCambia2300-*MdATG18a* plasmid; M, molecular marker DL2000. (b) Southern blot of tomato transgenic lines. Lanes: M, Maker; WT, wild type ‘Micro-Tom’ plants; OE-1~9, nine transgenic lines; V, positive vector containing pCambia2300-*MdATG18a* plasmid. (c) Southern blot of apple transgenic lines. Lanes: M, Maker; WT, non-transformed wild-type; OE-3 and -11, *MdATG18a*-transgenic lines; V, positive vector containing pCambia2300-*MdATG18a* plasmid.
